# PEG-Coated MnZn Ferrite Nanoparticles with Hierarchical Structure as MRI Contrast Agent

**DOI:** 10.3390/nano13030452

**Published:** 2023-01-22

**Authors:** Sedigheh Cheraghali, Ghasem Dini, Isabella Caligiuri, Michele Back, Flavio Rizzolio

**Affiliations:** 1 Department of Molecular Sciences and Nanosystems, Ca’ Foscari University of Venice, 30172 Venice, Italy; 2 Department of Nanotechnology, Faculty of Chemistry, University of Isfahan, Isfahan 81746-73441, Iran; 3 Pathology Unit, Centro di Riferimento Oncologico di Aviano (CRO) IRCCS, 33081 Aviano, Italy

**Keywords:** MnZn ferrite, nanoparticles, hierarchical nanostructure, MRI contrast agent, PEGylation process

## Abstract

In this work, MnZn ferrite nanoparticles with hierarchical morphology were synthesized hydrothermally, and their surface characteristics were improved by the PEGylation process. In vitro MRI studies were also conducted to evaluate the ability of the synthesized nanoparticles as a contrast agent. All results were compared with those obtained for MnZn ferrite nanoparticles with normal structure. Microstructural evaluations showed that in ferrite with hierarchical morphology, the spherical particles with an average size of ~20 nm made a distinctive structure consisting of rows of nanoparticles which is a relatively big assembly like a dandelion. The smaller particle size and dandelion-like morphology led to an increase in specific surface area for the hierarchical structure (~69 m^2^/g) in comparison to the normal one (~30 m^2^/g) with an average particle size of ~40 nm. In vitro MRI, cytotoxicity and hemocompatibility assays confirmed the PEG-coated MnZn ferrite nanoparticles with hierarchical structure synthesized in the current study can be considered as an MRI contrast agent.

## 1. Introduction

Magnetic resonance imaging (MRI), a common non-invasive imaging method in molecular medicine for diagnosing diseases, is commonly used to scan soft tissues (mainly malignancies) and ensure the availability for the precise examination of the discrepancies between healthy and cancerous cells. High resolution, non-ionizing radiation, and precise anatomical area delineation seem to be just a few advantages of this method. However, some efforts have been performed to ameliorate its inherent limitations, specifically the low signal sensitivity as a significant constraint [1,2,3].

High-resolution magnetic resonance images can be produced using contrast agents. Over 300 million doses of gadolinium-based contrast agents (GBCAs) as T_1_ positive-contrast agents have been used worldwide for approximately 30 years. Very few gadolinium-related toxicities have been documented and these agents have an outstanding safety profile overall; however, nephrogenic systemic fibrosis (NSF), a rare but serious disease found in patients with severe renal failure has been reported for GBCAs [4,5].

Superparamagnetic nanoparticle synthesis has grown significantly, in part due to its scientific utility, which includes magnetic storage media, biosensors, and medical applications such as drug carriers, T_2_ negative-contrast agents for MRI, etc. Because the characteristics of nanocrystals greatly depend on the size of nanoparticles, controlling the monodisperse size is crucial. Compared to conventional paramagnetic MRI contrast agents based on gadolinium compounds, ferrite (Fe_3_O_4_) nanoparticles exhibit excellent magnetization and longer circulation times [6,7]. The most attractive ferrites are multi-substituted ones because of their potential for medical diagnosis and treatment [8,9]. The magnetic properties in these ferrite nanoparticles depend on the type and the percentages of each component along with the particle size and colloidal stability. Comparing different ferrite nanoparticles has indicated that manganese-zinc (MnZn) ferrite nanoparticles with a chemical composition of Mn_0.5_Zn_0.5_Fe_2_O_4_ are a great choice as an MRI contrast agent because of their high saturation magnetization (Ms) and low coercivity compared to ferrite nanoparticles and other oxides [8,9,10,11]. In addition, the improvement of the magnetic properties of ferrite nanoparticles via the synthesis method by controlling the processing parameters may result in a reduction in the proportion of nanoparticles required for MRI applications [11]. For example, hydrothermal synthesis is an interesting technique, particularly for biomedical fields, because the features of produced powder can be easily controlled by processing parameters such as pH, time, and the temperature of the reaction [12,13,14,15].

On the other hand, ferrite nanoparticles frequently require to be coated with biocompatible materials for successful MRI applications to stabilize their dispersions in a liquid under physiological circumstances. In order to achieve colloidal stability, a variety of natural and synthetic compounds, including polyethylene glycol (PEG), dextran, chitosan, and oleic acid have been utilized as a coating on the surface of nanoparticles [16,17,18]. The most widely used polymer among them is PEG. The low toxicity and immunogenicity of PEG, together with its hydrophilic property, make the PEG-coated nanoparticles undetectable to the defense mechanism. The PEG-coated nanoparticles are appealing for biological applications due to these characteristics [18,19].

As far as is known, although there are several studies on the hydrothermal synthesis of individually dispersed MnZn ferrite nanoparticles as MRI contrast agents [10,11,12], none of these studies investigated the efficacy of these nanoparticles as MRI contrast agents with a hierarchical structure. Hierarchical structures are assemblies of nanomaterials glued together, forming various morphologies, and leading to the formation of multifunctional materials with unique properties for different applications. In other words, the production of three-dimensional hierarchical nanostructures results from the right organization of various nanomaterials as building blocks with two or more levels ranging from the nanoscale to the macroscopic size. These structures have new uses in biology, environmental protection, material science, and other fields. The concept of hierarchical nanostructures has gained popularity and will be actively researched in the upcoming years [20,21].

Therefore, the purpose of this study was to explore the possibility of employing MnZn ferrite nanoparticles with hierarchical morphology produced hydrothermally as MRI contrast agents. A hierarchically MnZn ferrite structure was synthesized and then physically coated with PEG polymer. In vitro MRI studies were conducted to evaluate the ability of the synthesized nanoparticles as an MRI contrast agent. The toxicity and hemocompatibility of the products were also examined. All the results were compared with the results obtained for MnZn ferrite nanoparticles with a normal structure (i.e., individually dispersed nanoparticles).

## 2. Materials and Methods

### 2.1. Materials

Materials such as ferric chloride (FeCl_3_·6H_2_O), zinc chloride (ZnCl_2_), manganese chloride (MnCl_2_·4H_2_O), ascorbic acid (C_6_H_8_O_6_), urea (CO(NH_2_)_2_), ammonia solution (NH_4_OH, 25%), phosphate-buffered saline (PBS), and the PEG (MW = 6 kDa) were purchased from Merck Co. Double distilled water (DDW) was also used for all procedures.

### 2.2. Synthesis of MnZn Ferrites

Hierarchically MnZn ferrite powder with the chemical composition of Mn_0.5_Zn_0.5_Fe_2_O_4_ was synthesized according to the procedure proposed by Budhiraja [22] via hydrothermal technique. First, 3.24 g FeCl_3_·6H_2_O, 0.65 g ZnCl_2_, and 0.97 g MnCl_2_·4H_2_O were dissolved in 50 mL DDW, and then 3.17 g C_6_H_8_O_6_, and 3.3 g CO(NH_2_)_2_ were added. The prepared solution was stirred at room temperature for 30 min and then poured into a 200 mL-Teflon-lined stainless-steel autoclave and the reactor was kept in an electrical oven for 6 h at 160 °C. The resulting precipitates were centrifuged at 10,000 rpm and then washed several times until the pH of the suspension reached the pH of DDW (~6.5). Finally, the product was dried for 10 h at 70 °C followed by annealing for 2 h at 500 °C in an electrical furnace.

MnZn ferrite with identical composition and normal morphology was also synthesized according to Ref. [23]. For this reason, 3.24 g FeCl_3_·6H_2_O, 0.65 g ZnCl_2_, and 0.97 g MnCl_2_·4H_2_O were dissolved in 30 mL DDW. The pH of the solution was adjusted to 10 by dropwise adding NH_4_OH. The homogeneous solution was then transferred to the autoclave, and it was kept at 180 °C in the electric oven for 3 h. After cooling to room temperature, the product was collected and rinsed several times with DDW until the pH of the suspension reached the pH of DDW (~6.5).

### 2.3. Coating of PEG Polymer on MnZn Ferrites

To coat MnZn ferrite powders with PEG by physical bonding method, 0.5 g of both products (i.e., normal and hierarchical morphologies) was first dispersed in 50 mL of DDW at room temperature for 30 min via an ultrasonic bath. Also, 2 g of PEG was poured into 50 mL of DDW and stirred for 1 h. Then, this solution was added to the previous suspensions and stirred for 24 h. After separation by centrifugation, the coated products were dried at room temperature for 1 day.

### 2.4. Characterization

X-ray diffraction (XRD) patterns were recorded using an Asenware AW-DX 300 diffractometer (Dandong, China, λ = 1.54184 Å). MAUD program [24] was used to determine the phase content of each powder via the Rietveld refinement. A scanning electron microscope (SEM, ZEISS SIGMA 500 VP, Germany) and a transmission electron microscope (TEM, ZEISS EM10C, Germany) were used to investigate the morphology and particle size of the synthesized powders. Fourier-transform infrared spectroscopy (FTIR, JASCO-6300, Japan) and a carbon, hydrogen, nitrogen, sulfur (CHNS) elemental analyzer (Leco 932) were used to confirm the presence of PEG polymer around the particles and measured its content, respectively. Additionally, an inductively coupled plasma optical-emission spectrometer (ICP-OES, Perkin-Elmer Optima 7300 DV, USA) and an energy dispersive X-ray spectrometer (EDS, Oxford Instruments, UK) available in the SEM system were used to evaluate the Fe, Mn, and Zn concentrations of the produced particles. A Horiba SZ-100 series analyzer based on dynamic light scattering (DLS) was used to evaluate the stability of dispersions (~1 mg/mL) of the synthesized powders in PBS (pH = ~7.4). A vibrating sample magnetometer (VSM, Meghnatis Daghigh Kavir Co., Kashan, Iran) was used to evaluate the magnetic features of the PEG-coated and uncoated MnZn ferrites at room temperature. The specific surface area of the powders was determined via the Brunauer–Emmett–Teller (BET) method by adsorption/desorption of N_2_ gas at liquid nitrogen temperature (~77 K) using a Series BEL SORP mini II. A 1.5 T clinical MRI system (Philips, Ingenia) with repetition time = 3 s, 32 echoes with 110 ms, acquisition time of 5 min, field of view = 200 × 200 mm^2^, matrix size = 128 × 128 mm^2^, slice thickness = 3 mm was used for in vitro MRI tests to investigate the efficacy of the synthesized MnZn ferrites as an MRI contrast agent. In order to achieve this, colloids containing products at concentrations of 1 to 4 mM of metal (i.e., Mn + Zn + Fe) were prepared and poured into a 24-well plate placed in the iso-center of the magnet. Then, T_2_-weighted MR images of colloids were recorded. T_2_ relaxation time was measured by the Carr-Purcell-Meiboom-Gill (CPMG) sequence at room temperature and results were inverted to obtain the R_2_ relaxation rates in s^−1^. The effectiveness of each contrast agent was expressed in terms of T_2_ relaxivity, which is denoted as r_2_ [mM^−1^s^−1^]. Then, according to the slopes of relaxation rate curves and the concentration of all metal components, r_2_ was calculated (i.e., ΔR_2_ = R_2_ − R_2, 0_ = 1/T_2_ − 1/T_2, 0_, and T_2, 0_ = 367 ms was the relaxation time of pure PBS solution).

### 2.5. Complete Blood Count (CBC) Test

Blood samples from a healthy volunteer were mixed with different powders to create colloids at concentrations of 0.1, 0.2, and 0.3 mg/mL. Then, using a Mindray BC-6800 automated hematology analyzer, the red blood cell (RBC) count of the colloids was determined and compared with the blood sample without nanoparticles as the control sample.

### 2.6. In Vitro Blood Coagulation Test

First, a platelet-poor plasma (PPP) was obtained by centrifuging the blood sample at 3000 rpm. PPP was then mixed with the powders to create colloids at concentrations of 0.1, 0.2, and 0.3 mg/mL. Prothrombin time (PT) and activated partial thromboplastin time (*a*PTT), two blood coagulation factors of the colloids, were tested using an automation blood coagulation analyzer (STA Compact Stago, USA) and results were compared with PPP without nanoparticles as the control sample.

### 2.7. Cytotoxicity Assay

The cytotoxicity test was carried out for 96 h on MRC5 (normal fibroblast), OVCAR3, Kuramochi (ovarian cancer), and DLD-1 (colon cancer) cell lines. Cells were plated on 96 multiwells and treated with a serial dilution of nanoparticles starting from 1 × 10^−2^ to 1 × 10^3^ μg/mL. The viability was evaluated with CellTiter-Glo^®^ Luminescent Cell Viability Assay in a Tecan M1000 instrument. Data were analyzed with a nonlinear regression (curve fit) model with Prism software.

### 2.8. Statistical Analysis

SPSS software (version 22) was used for statistical analysis. Significant differences were identified by one-way analysis of variance (ANOVA) and the multiple-comparison (Tukey’s) test with n = 3. The results were reported as mean ± standard deviation (SD) with a significant value of *p* < 0.05.

## 3. Results and Discussion

**Figure 1:**
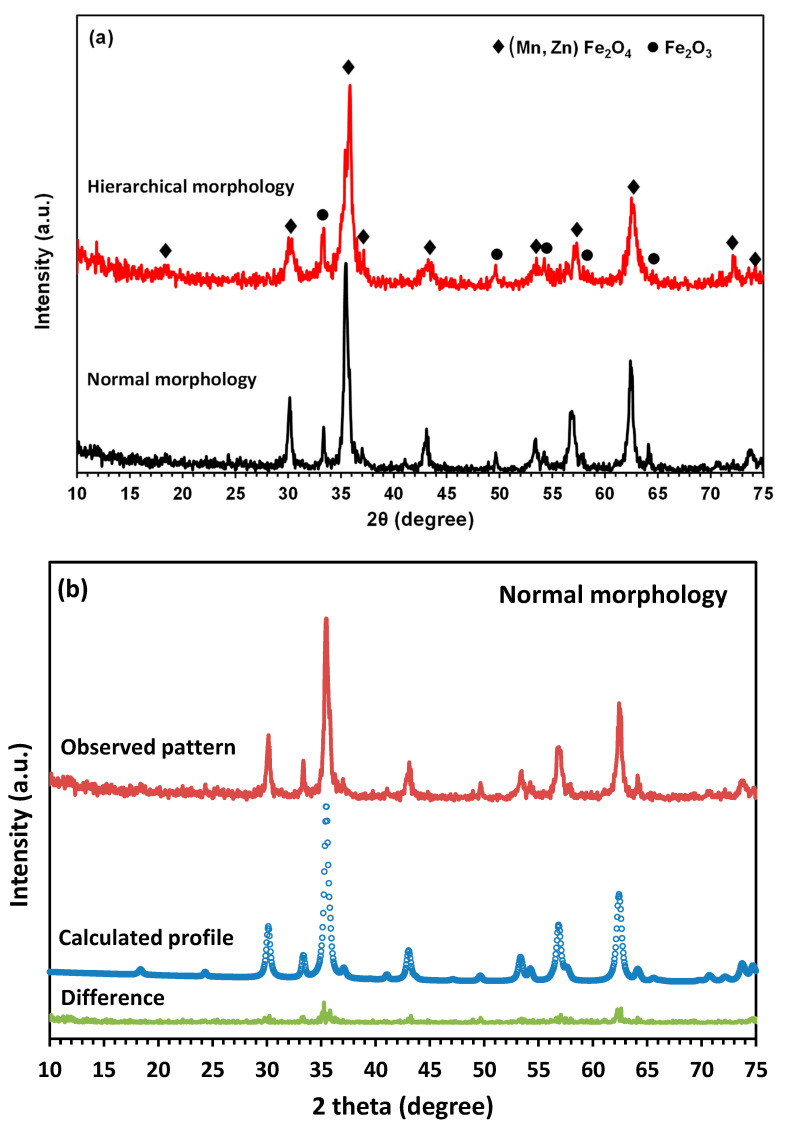

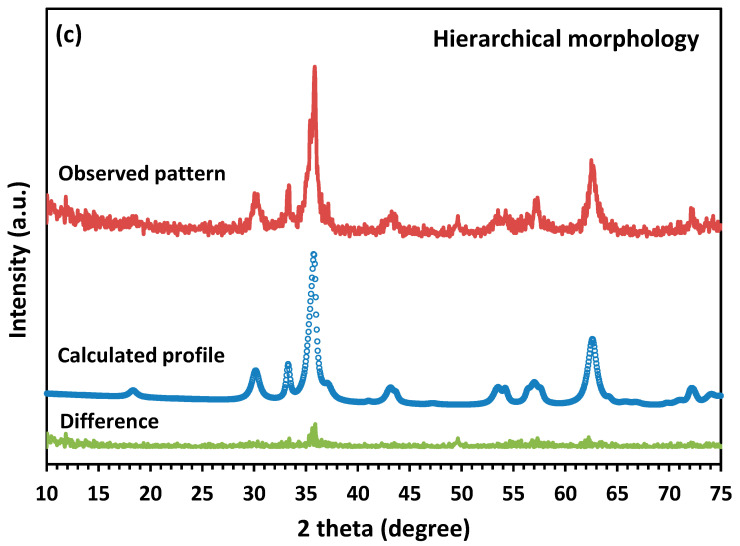
(**a**) XRD patterns of synthesized MnZn ferrites with different morphologies, and (**b**), and (**c**) corresponding Rietveld refined XRD patterns.

The XRD patterns of the synthesized powders are shown in Figure 1a. In both products, all the major peaks are corresponding to the cubic structure of (Mn, Zn) Fe_2_O_4_ (ICDD PDF no. 01-074-2401). Although some minor peaks can be assigned to Fe_2_O_3_ (ICDD PDF no. 01-089-0597), according to the quantitative phase analysis via the Rietveld refinement (Figure 1a,b), the content of this structure in both synthesized powders is less than 10 wt.% (i.e., 5 wt.% in MnZn ferrite with normal morphology, and 7 wt.% in MnZn ferrite with hierarchical morphology). The same results have been presented elsewhere and it has been suggested that the purity of the synthesized ferrite could be improved by optimization of the hydrothermal parameters such as time and temperature [25,26].

**Figure 2:**
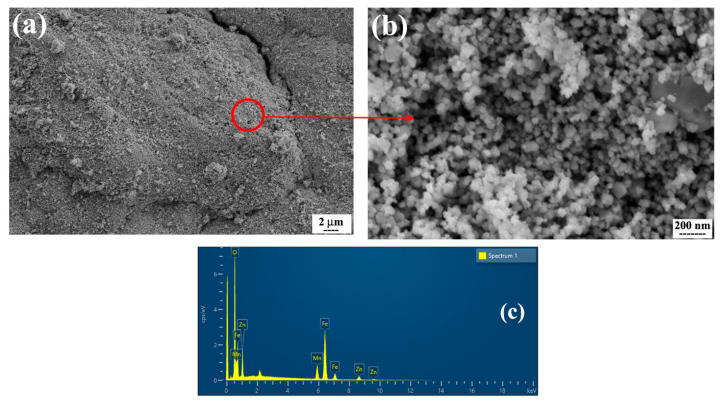
(**a**,**b**) SEM micrographs of synthesized MnZn ferrite with normal morphology at different magnifications, and (**c**) corresponding EDS results.

**Figure 3:**
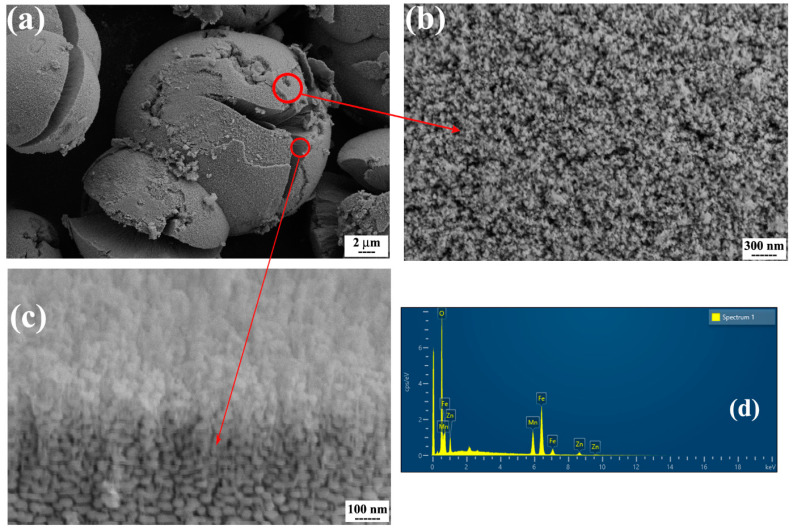
(**a**–**c**) SEM micrographs of synthesized MnZn ferrite with hierarchical morphology at different magnifications, and (**d**) corresponding EDS results.

Figure 2 and Figure 3 show the SEM micrographs and corresponding EDS results of ferrites with normal and hierarchical morphology. In both powders, the particles are almost spherical and have a relatively uniform distribution. The SEM images also show that the average size of the particles is on the nanoscale. However, it clearly can be seen that the arrangement of the spherical nanoparticles is completely different in both powders. Although ferrite with normal morphology consists of individual nanoparticles, in ferrite with hierarchical morphology, the particles make a distinctive structure consisting of rows of nanoparticles which is a relatively big assembly like a dandelion on the micron scale. It has been suggested that the presence of ascorbic acid and urea leads to a special arrangement of nanoparticles in various directions during the hydrothermal synthesis of this powder [22]. Moreover, the EDS results (Figure 2c and Figure 3d) show that in both powders, Fe, Mn, Zn, and O elements are present. The ICP data (Table 1) also shows that both synthesized MnZn ferrites have approximately designed stoichiometric ratios for the Mn, Zn, and Fe elements.

**Figure 4:**
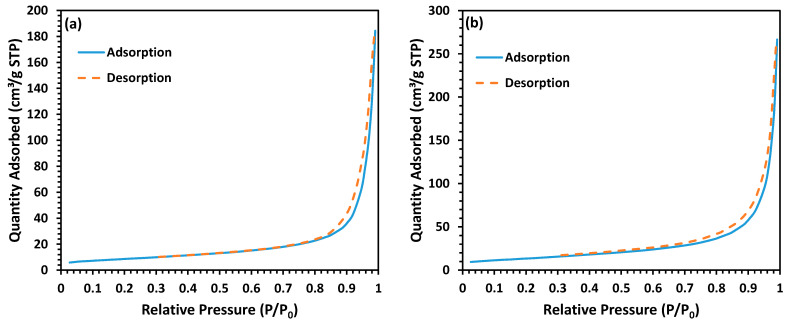
N_2_ adsorption/desorption isotherms at 77 K for MnZn ferrite nanopowders with (**a**) normal morphology, and (**b**) hierarchical morphology.

One of the key characteristics that demonstrates a material’s capacity to interact with its environment is surface area. A bigger surface area allows the material to absorb more molecules, which improves performance significantly in applications such as drug release activated by alternating magnetic fields [27]. A decrease in particle size leads to increasing the surface area, and as a direct consequence, different arrangements of nanoparticles in a hierarchical 3D nanostructure could significantly affect the textural properties of the material [21,27]. The N_2_ adsorption-desorption isotherms (Figure 4) confirmed that the MnZn ferrite with hierarchical morphology has a relatively high BET surface area (~69 m^2^/g) in comparison to its counterpart (~30 m^2^/g) with normal morphology. This enhancement provides some useful characteristics for MnZn ferrite with hierarchical morphology such as higher drug loading capacity for drug delivery applications.

**Figure 5:**
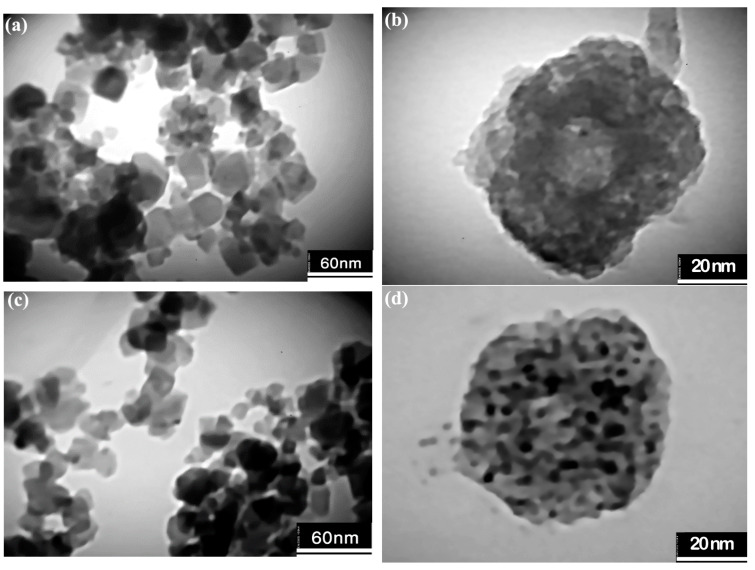
TEM micrographs of bare and PEG-coated MnZn ferrite nanoparticles, (**a**,**b**) with normal morphology, and, (**c**,**d**) with hierarchical morphology.

**Figure 6:**
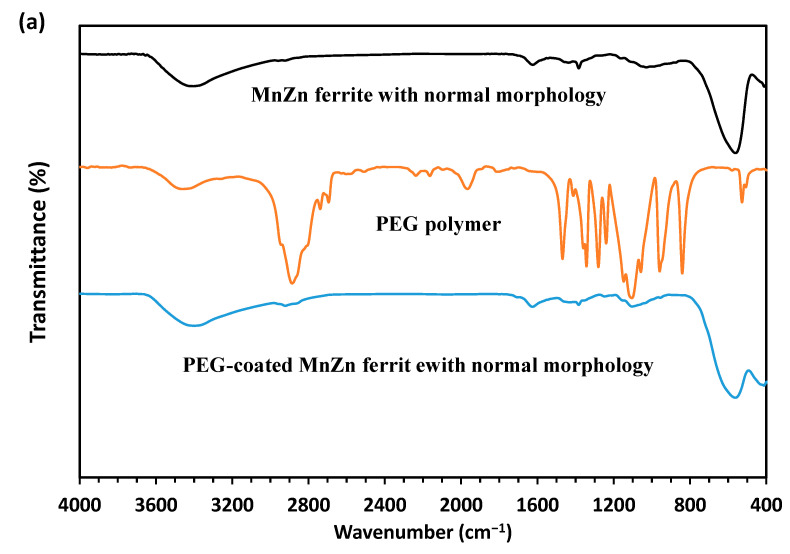

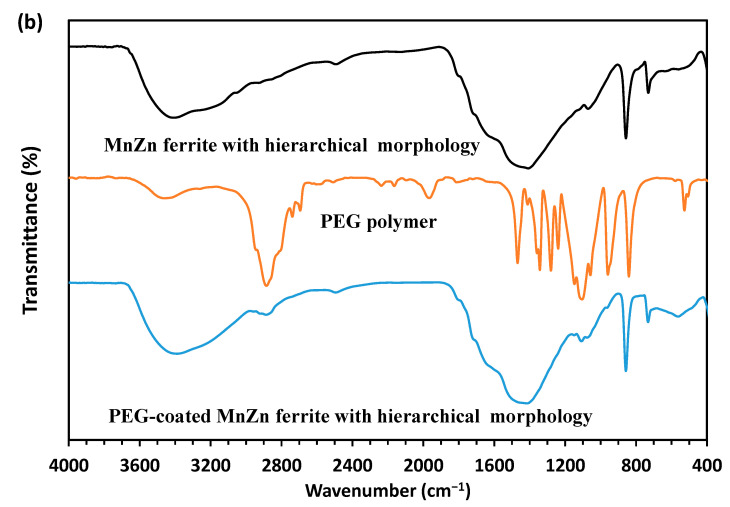
(**a**,**b**) FTIR spectra of bare, PEG-coated MnZn ferrite nanoparticles with different morphologies.

To better study the shape and size of the synthesized nanoparticles and also to confirm the presence of PEG coating, TEM analysis was performed and the results are presented in Figure 5. As can be seen from Figure 5a,c, the average sizes of semi-spherical nanoparticles of MnZn ferrite with normal and hierarchical morphologies are about 40 and 20 nm, respectively. PEG coating has also appeared as a light gray substance around the clusters of nanoparticles in both powders (Figure 5b,d). Since PEG-coated nanoparticles must be dried for TEM analysis, so the particles were slightly agglomerated. Additionally, FTIR analysis was used to identify PEG in coated-ferrite powders. The FTIR spectra of bare nanoparticles, PEG polymer as well as coated nanoparticles are illustrated in Figure 6a,b. In the FTIR spectra of PEG, the -C-O-C- bending vibration peak may be found near 1100 and 1350 cm^−1^, respectively. Additionally, the absorption peaks at 950 cm^−1^ are due to the out-of-plane bending vibration of -CH, whereas the peaks around 1280 and 1470 cm^−1^ are due to the bending vibrations of -CH2 [15,18,19]. Furthermore, the main absorbance of the ether stretch and the vibrational peaks that are seen in the FTIR spectrum of PEG is present in the FTIR spectrum of the PEG-coated MnZn ferrites with normal and hierarchical. In addition, the organic content in the bare and PEG-coated MnZn ferrite nanoparticles with different morphologies was determined by the CHNS analysis. The results showed that the organic content in the PEG-coated MnZn ferrite nanoparticles with normal and hierarchical morphologies was 18.5 and 20.1 wt.%, respectively, and only consisted of C and H elements. Moreover, there was no organic compound in the bare MnZn ferrite nanoparticles. Therefore, grey matter around both coated nanoparticles in corresponding TEM micrographs (i.e., Figure 5b,d) could be ascribed to the PEG polymer.

**Figure 7:**
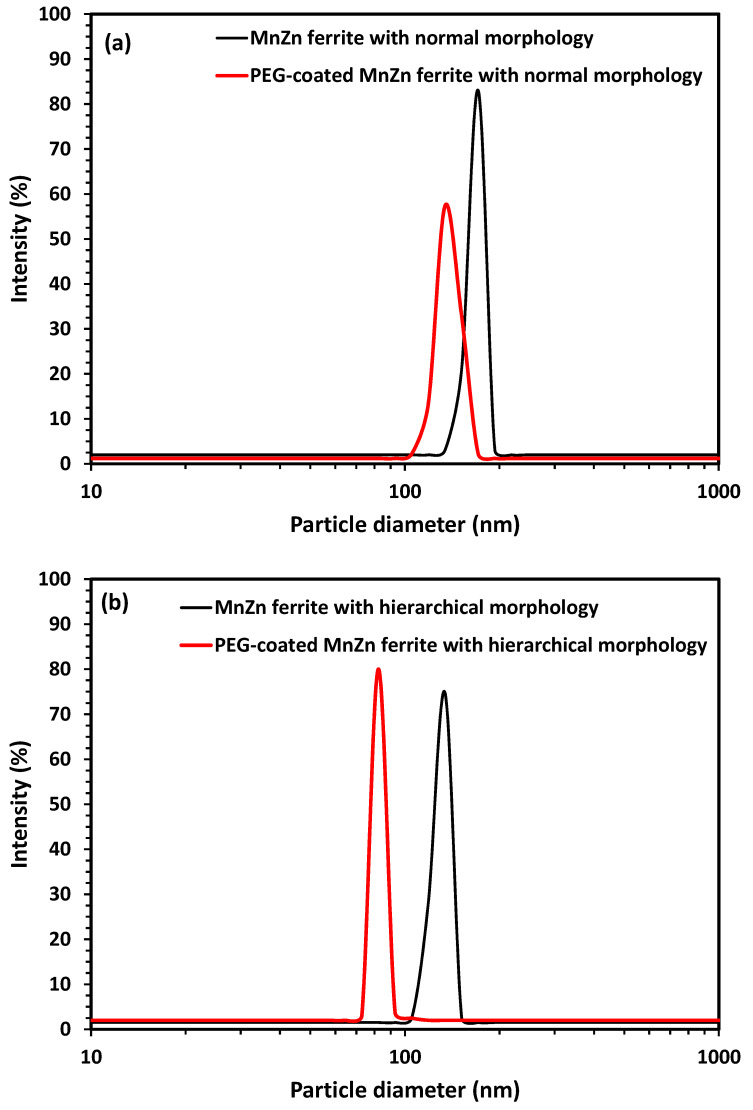

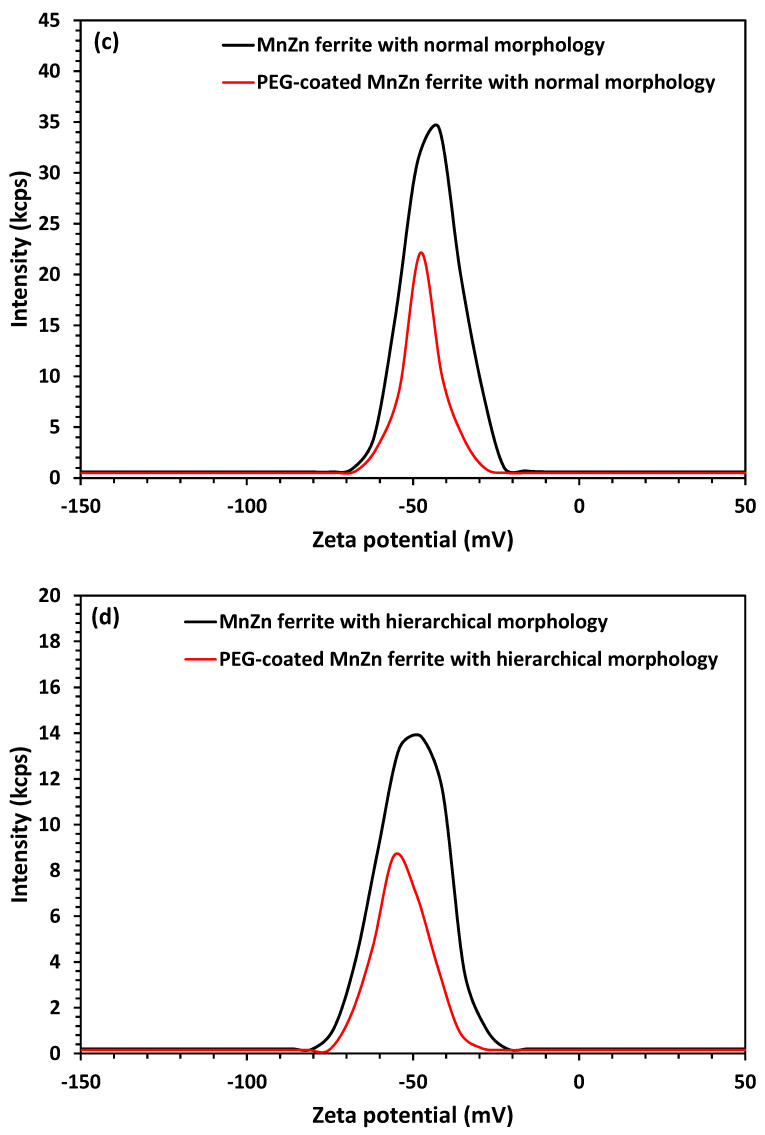

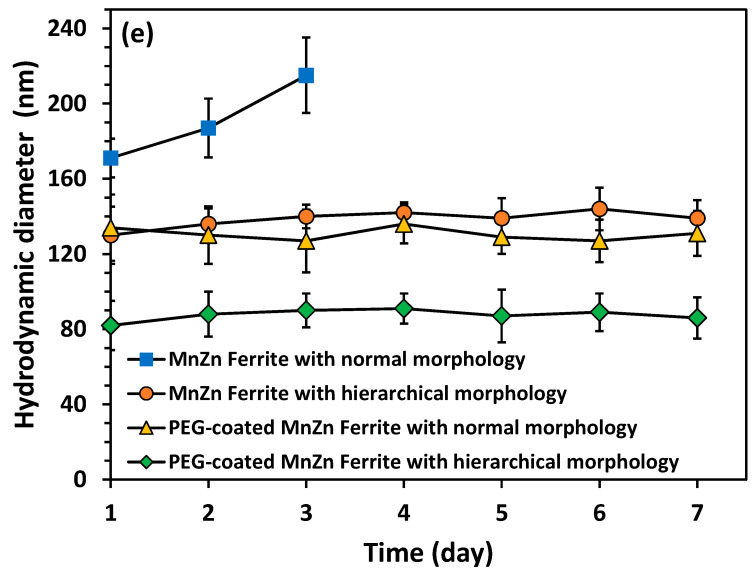
(**a**,**b**) Graphs of DLS, (**c**,**d**) graphs of zeta potential analyses, and (**e**) results of colloidal stability test for suspensions containing PEG-coated and uncoated MnZn ferrite nanoparticles with different morphologies. Error bars are standard deviation (n = 3).

Due to the high specific surface and also magnetic force between particles, ferrite powders could easily be aggregated. Therefore, coating nanoparticles with non-toxic, non-immunogenic polymers such as PEG (i.e., PEGylation process) is an effective way to improve the colloidal stability of dispersions containing ferrite nanoparticles in biological media and a magnetic field. The graphs of DLS and zeta potential analyses of colloids containing both synthesized MnZn ferrites with and without PEG coating are illustrated in Figure 7. Clearly, PEG coating not only leads to a decrease in the hydrodynamic diameter of nanoparticles as a sign of deagglomeration (i.e., 171.5 ± 10.3 to 133.9 ± 17.6 nm in ferrite with normal morphology, and 130.3 ± 15.2 to 81.8 ± 13.1 nm in ferrite with hierarchical morphology), but also the surface charge of coated nanoparticles shifted to lower values (i.e., −42.3 ± 2.3 to −47.4 ± 3.7 mV in ferrite with normal morphology, and −40.9 ± 2.1 to −55 ± 4.1 mV in ferrite with hierarchical morphology), improving colloidal stability [16,18,28,29]. To investigate the colloidal stability of the prepared suspensions, a new set of DLS experiments was conducted in which the hydrodynamic diameter of nanoparticles in each prepared suspension was measured for one week. The obtained results (Figure 7e) confirmed the PEG-coated MnZn ferrite nanoparticles with different morphologies and also the bare MnZn ferrite nanoparticles with hierarchical morphology remained stable in PBS medium over a week, while the bare MnZn ferrite nanoparticles with normal morphology started to sediment after three days.

In comparison to the TEM results, it should be noted that the observed difference in the average size obtained via the DLS and TEM methods is due to the hydrodynamic diameter of particles in the DLS measurements. This may result from the “hair layer” theory which is a reaction to the formation of a hairy layer due to molecular chains of PEG on the surface of nanoparticles [18].

**Figure 8:**
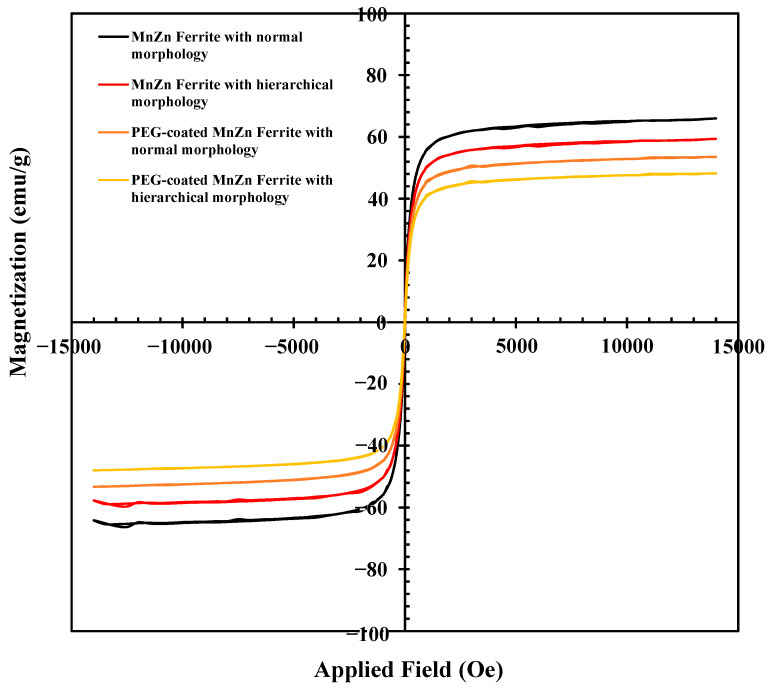
Magnetic hysteresis loops of the PEG-coated and uncoated MnZn ferrite nanoparticles with different morphologies.

Magnetic hysteresis loops of bare and coated MnZn ferrite nanoparticles with normal and hierarchical structures obtained by VSM are illustrated in Figure 8. Both materials exhibit superparamagnetic behavior (i.e., zero remanence and coercivity). In comparison to Ref. [11], all the synthesized powders in the current study exhibit relatively high Ms values (i.e., 66, 59, 54, and 48 emu/g for bare MnZn ferrite powders, and PEG-coated MnZn ferrite powders with normal and hierarchical morphologies, respectively) that are acceptable in terms of magnetic properties. However, Jang et al. [9] synthesized a series of (Zn_x_Mn_1−x_) Fe_2_O_4_ nanoparticles and they reported a maximum Ms of 175 emu/g for Zn_0.4_Mn_0.6_Fe_2_O_3_ nanoparticles. Although the hierarchical structure led to a decrease in Ms in bare MnZn ferrite nanoparticles (66 to 59 emu/g), its value is still significant. The decrease in Ms value for MnZn ferrite with hierarchical morphology could be attributed to its smaller particle size. The magnetic property and subsequent response to magnetic fields can be significantly influenced by particle size. For instance, the Ms value of ferrite particles drops as the particle size decreases, but the particles might still display supermagnetism behavior. As quantitative XRD analysis also confirmed, MnZn ferrite with hierarchical morphology contains a slightly higher content of Fe_2_O_3_ which may alter the corresponding Ms value. On the other hand, PEG as a polymer coating led to a decrease in Ms values of both coated ferrites. However, it is worth noting that coated ferrites still have a high MS. As mentioned before, the presence of PEG coating improves the stability of the colloid. In addition, according to the proposed method by Tenório-Neto et al. [30], the organic content in the PEG-coated nanoparticles was measured based on the Ms value of the bare nanoparticles and that of PEG-coated nanoparticles. This content was determined at about 19 wt.% for both PEG-coated MnZn ferrites with different morphologies and is almost consistent with those obtained by CHNS analyses, as previously mentioned.

**Figure 9:**
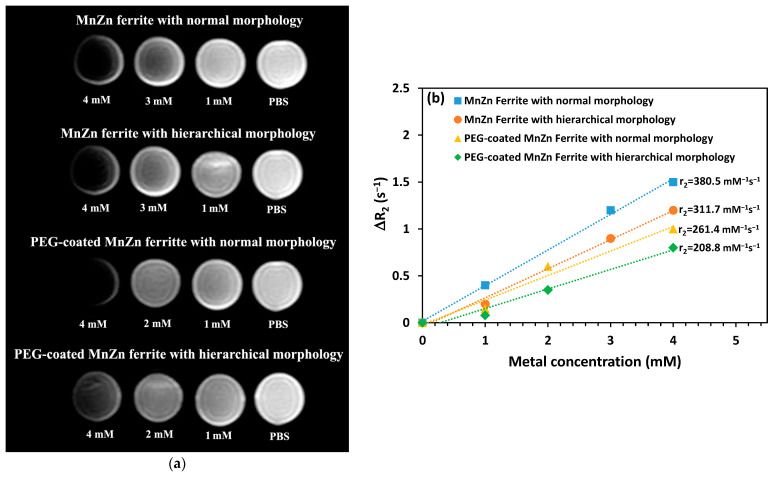
Effect of nanoparticle concentration on (**a**) in vitro T_2_-weighted MR images of PBS media containing PEG-coated and uncoated nanopowders with different morphologies, and (**b**) T_2_ relaxation rate against metal (i.e., Mn + Zn + Fe) concentration for the PEG-coated and bare MnZn ferrite NPs dispersions with different morphologies.

In the MRI technique, the contrast improvement directly corresponds to the Ms value of the contrast agent. Superparamagnetic nanoparticles such as MnZn ferrite as contrast agents could produce negative contrast images by reducing the signal intensity to produce dark images. In vitro T_2_-weighted MR images (Figure 9a) were taken from PBS media containing different concentrations of both the nanopowders with and without PEG coating and compared with PBS as the control. Figure 9a demonstrates that adding PEG-coated and uncoated MnZn ferrite nanoparticles to PBS as the control considerably improved the contrast quality of MR images. As can be predicted, this improvement got better as the nanoparticle concentration increased in both instances. However, the PEG-coated nanoparticle dispersions as well as bare nanoparticles with hierarchical structure exhibit slightly less improvement in the contrast of MR images than uncoated nanoparticles with normal structure. This result agrees well with the magnetic characteristics determined using VSM (Figure 8). The presence of the non-magnetic PEG coating on the surface of nanoparticles is often related to this, as was previously indicated. The contrasting impact of the produced MnZn ferrite nanoparticles is decreased as a result of this layer’s protection against protons [15]. Bare MnZn ferrite nanoparticles with normal morphology also showed the highest relaxivity of 380.5 mM^−1^s^−1^. In addition, the interesting result is that all the samples synthesized in this work exhibit higher relaxivity (Figure 9b) than reported values for some commercially approved MRI contrast agents of superparamagnetic iron oxide such as Feridex, Resovist, and Combidex at 1.5 T [31]. However, it must be noted that not only some parameters of ferrite nanoparticles such as the crystallinity, composition, size, morphology, and magnetic properties are responsible for T_2_ contrast but also the characteristics of nonmagnetic polymers as coatings must be considered. Cho et al. [32] have recently reported that higher T_2_ relaxivity could be achieved by manipulating the features of surface coating and ferrite nanoparticles together.

**Figure A1:**
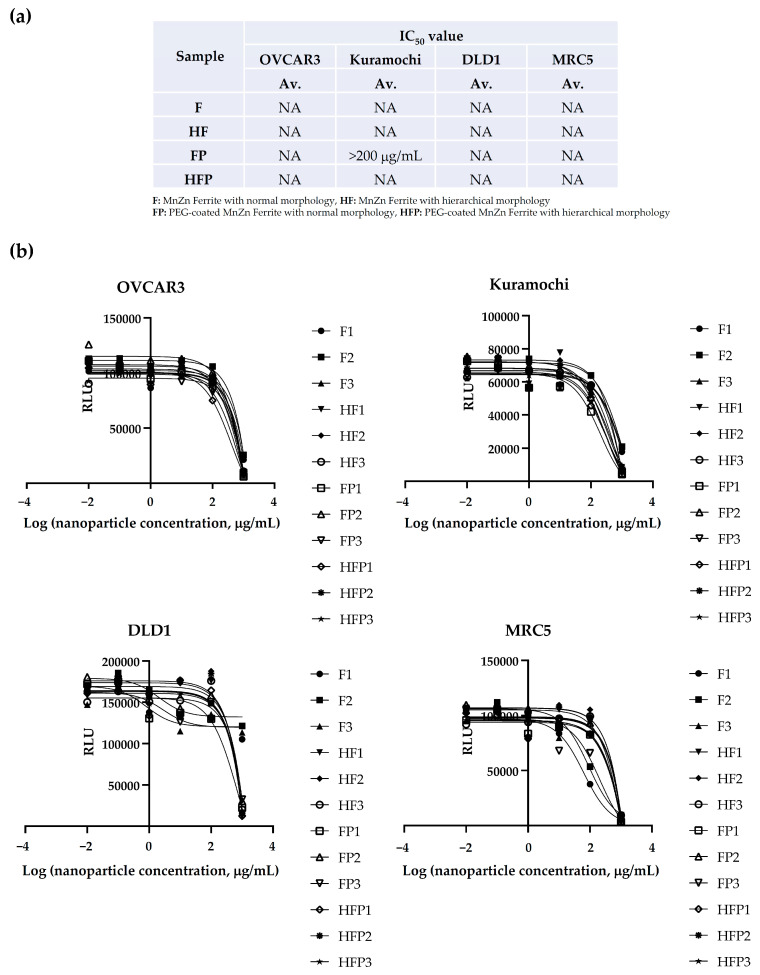
(**a**) Summary of the data presented in b. Values are expressed as µg/mL. Av: average, (**b**) Nonlinear regression analysis of different cells treated with nanoparticles in triplicates. On the Y axis, the relative luminescent values (RLU) are reported. On the X axis, the concentration of nanoparticles on a logarithmic scale is reported.

The results of RBC counts obtained from CBC tests, and also *a*PPT and PT values as coagulation factors for the blood samples treated with PEG-coated and uncoated MnZn ferrite nanoparticles with normal and hierarchical structures at concentrations of 0.1, 0.2, and 0.3 mg/mL are given in Table 2. The statistical analysis showed no significant difference (*p* < 0.05) in all cases from the corresponding control sample. So, it can be concluded that neither the PEG-coated nor the uncoated synthesized MnZn nanoparticles show any procoagulant activity at the tested doses, preventing their usage in intravenous applications. The biocompatibility of the synthesized products was even tested on normal and cancer cell lines but the IC_50_ values were very high (>0.2 mg/mL), indicating low cytotoxicity (Appendix A, Figure A1).

## 4. Conclusions

In this work, the hydrothermal method was used to synthesize MnZn ferrite nanoparticles with hierarchical assembly. The effect of the PEGylation process on the magnetic properties and biocompatibility of the synthesized nanoparticles was also studied. All the results were compared with those obtained for MnZn ferrite nanoparticles with normal structure (i.e., individual particles). The following results were obtained in this study:

1. The average particle size of the synthesized MnZn ferrites with normal and hierarchical structures was about 40, and 20 nm, respectively.
2. PEG coating improved the colloidal stability and biocompatibility of nanoparticles with a slight decrease in Ms values.
3. Adding PEG-coated and uncoated MnZn ferrite nanoparticles to PBS considerably improved the contrast quality of MR images.
4. RBC, blood coagulation and cell cytotoxic studies under laboratory conditions showed that both synthesized MnZn ferrite nanoparticles have no negative effects on blood factors and cell viability, respectively.
5. The PEG-coated MnZn ferrite nanoparticles with hierarchical structure synthesized in the current study can be considered as an MRI contrast agent at concentrations between 0.1 and 0.3 mg/mL.

## Figures and Tables

**Table 1:** ICP date for both synthesized ferrite powders.

Powder	Fe (wt.%)	Mn (wt.%)	Zn (wt.%)	Molar Ratio (Fe/Mn + Zn)
Ferrite with normal morphology	65.1	18.3	16.6	1.99
Ferrite with hierarchical morphology	63.9	17.6	18.5	1.90

**Table 2:** Effect of concentration and morphology of MnZn ferrite nanoparticles on values of RBC, *a*PPT, and PT of blood samples.

Powder	Concentration (mg/mL)	RBC * (10^6^/µL)	*a*PPT * (s)	PT * (s)
MnZn ferrite with normal morphology	0.1	5.37 ± 0.02	30.1 ± 0.1	12.9 ± 0.1
	0.2	5.48 ± 0.02	30 ± 0.2	13.0 ± 0.2
	0.3	5.40 ± 0.03	29.5 ± 0.1	12.9 ± 0.2
PEG-coated MnZn ferrite with normal morphology	0.1	5.32 ± 0.05	29.8 ± 0.3	13.1 ± 0.3
	0.2	5.51 ± 0.02	30.4 ± 0.2	12.9 ± 0.3
	0.3	5.48 ± 0.04	29.9 ± 0.2	12.9 ± 0.2
MnZn ferrite with hierarchical morphology	0.1	5.39 ± 0.03	31.4 ± 0.1	13.1 ± 0.2
	0.2	5.42 ± 0.02	30 ± 0.3	12.9 ± 0.2
	0.3	5.52 ± 0.02	30.2 ± 0.2	12.9 ± 0.3
PEG-coated MnZn ferrite with hierarchical morphology	0.1	5.38 ± 0.04	29.5 ± 0.3	12.8 ± 0.1
	0.2	5.47 ± 0.04	29.6 ± 0.3	12.8 ± 0.2
	0.3	5.48 ± 0.03	29.9 ± 0.2	12.9 ± 0.2
Control	-	5.62 ± 0.05	31.4 ± 0.2	12.8 ± 0.3

* Mean values are not significantly different (*p* < 0.05). Errors are standard deviation (n = 3).

## Data Availability

The data presented in this study are available on request from the corresponding author.
